# Oxidative stress in the bladder of men with LUTS undergoing open prostatectomy: a pilot study

**DOI:** 10.1590/S1677-5538.IBJU.2018.0127

**Published:** 2018

**Authors:** Marcio Augusto Averbeck, Nelson Gianni de Lima, Gabriela Almeida Motta, Lauro Beltrão, Nury Jafar Abboud, Clarice Pereira Rigotti, William Nascimento dos Santos, Steven Kitzberger Jaeger dos Santos, Luis Fernando Batista da Silva, Ernani Luis Rhoden

**Affiliations:** 1 Departamento Pós-graduação em Ciências da Saúde da Universidade Federal de Ciências da Saúde de Porto Alegre (UFCSPA), Porto Alegre, RS, Brasil; 2 Serviço de Urologia, Complexo Hospitalar Santa Casa, Porto Alegre, RS, Brasil; 3 Serviço de Urologia, Hospital Moinhos de Vento, Porto Alegre, RS, Brasil; 4 Serviço de Urologia, Hospital de Nossa Senhora dos Navegantes, Torres, RS, Brasil; 5 Disciplina de Urologia, Universidade Federal de Ciências da Saúde de Porto Alegre (UFCSPA), Porto Alegre, RS, Brasil

**Keywords:** Urinary Bladder, Lower Urinary Tract Symptoms, Urinary Bladder Neck Obstruction, Oxidative Stress

## Abstract

**Purpose:**

This study aims to evaluate the link between preoperative parameters and oxidative stress (OS) markers in the bladder wall of men undergoing open prostatectomy.

**Materials and Methods:**

From July 2014 to August 2016, men aged ≥ 50 years and presenting with LUTS were prospectively enrolled. Preoperative assessment included validated questionnaires (IPSS and OAB - V8), lower urinary tract ultrasound and urodynamics. Bladder biopsies were taken during open prostatectomy for determination of OS markers. Increased OS was defined by increased concentration of malondialdehyde (MDA) and / or decreased concentration of antioxidant enzymes (superoxide dismutase and / or catalase). P<0.05 was regarded as statistically significant.

**Results:**

Thirty - eight consecutive patients were included. Mean age was 66.36 ± 6.44 years, mean prostate volume was 77.7 ± 20.63 cm3, and mean IPSS was 11.05 ± 8.72 points. MDA concentration was increased in men with severe bladder outlet obstruction (BOO grade V - VI according to the Schaefer's nomogram) in comparison with BOO grade III - IV (p = 0.022). Patients with severe LUTS also had higher MDA concentration when compared to those with mild LUTS (p = 0.031). There was a statistically significant association between increased post - void residual urine (cut off ≥ 50 mL) and not only higher levels of MDA, but also reduced activity of SOD and catalase (p < 0.05).

**Conclusions:**

This pilot study showed that severity of LUTS and BOO were associated with increased MDA concentration in the bladder wall of men undergoing open prostatectomy. Further studies are still needed to assess the role of non - invasive biomarkers of OS in predicting bladder dysfunction in men with LUTS.

## INTRODUCTION

Current evidence suggests that bladder response to chronic obstruction occurs adaptively (1). Bladder functional changes caused by obstruction may be urodynamically classified in three main groups: (a) detrusor overactivity with or without reduced bladder compliance; (b) detrusor underactivity (DU) with impaired voiding; and (c) mixed pattern (2).

Unfortunately, to date there is no reliable marker to predict which patients with bladder outlet obstruction (BOO) will inexorably present deterioration of bladder contractility, which, by itself, has been associated with poorer surgical outcomes in men with benign prostatic hyperplasia (BPH). Thomas et al. demonstrated the lack of long - term symptomatic or urodynamic gains from transurethral resection of the prostate (TURP) in men with both BPH and detrusor underactivity (3). On the other hand, persistent detrusor overactivity is also clinically relevant in patients undergoing prostate surgery, as it may impose increased risk of urgency urinary incontinence (4).

According to animal models, oxidative stress (OS) and bladder dysfunction (BD) may be related to ischemia - reperfusion process and BOO (5, 6). Reactive oxygen species (ROS), including hydroxyl radicals, superoxide anions, and hydrogen peroxide, are normally produced in low levels during univalent reduction of oxygen to water and are important for diverse biological processes, including apoptosis, immunity, and cell defense against microorganisms (7). Increased formation of ROS and / or decreased antioxidant defense can be defined as OS, which may cause cell damage. Endogenous antioxidant enzymes such as superoxide dismutase (SOD) and catalase (CAT) are key to prevent damage from ROS. OS induces lipid peroxidation, which is expressed by formation of malondialdehyde (MDA) (8). Sezginer et al. has recently investigated the effects of different degrees of obstruction on bladder function in rats, showing that MDA levels were increased in severe partial BOO (9). Nevertheless, this association has not been properly assessed in humans so far.

Our study aims to evaluate the link between preoperative parameters (clinical, ultrasound and urodynamic findings) and OS markers in the bladder wall of men undergoing open prostate surgery. We hypothesized that severe bladder outlet obstruction was associated with increased OS in the bladder wall of men with lower urinary tract symptoms (LUTS).

## MATERIALS AND METHODS

This was a pilot study, approved by the local Ethics Committee (approval number: 660.810). From July 2014 to August 2016, men presenting with LUTS, aged ≥ 50 years, prostate volume ≥ 40 mL, undergoing prostate surgery in a single university hospital were invited to take part in the study. The target population consisted of patients with BPH or organ - confined prostate cancer with concomitant LUTS in the perioperative period of open prostatectomy.

Our primary endpoint was the association between OS markers and severity of BOO. Exploratory endpoints included the link between OS markers various preoperative characteristics, such as obesity, severity of LUTS, overactive bladder symptoms, ultrasound and urodynamic parameters.

Exclusion Criteria: Patients without complaint of one or more voiding LUTS over the last 3 months, previous pelvic surgery, neurological disease with secondary neurogenic lower urinary tract dysfunction, established cardiovascular disease (including prior stroke, myocardial ischemia and / or peripheral vascular disease) and patients relying on clean intermittent catheterization or taking drugs with potential effects on bladder function (e.g. anticholinergics and 5 - alpha - reductase inhibitors).

Patients who met the criteria for inclusion were invited to participate and received comprehensive information on further evaluations, which included lower urinary tract ultrasound, urodynamics and a bladder wall biopsy. Only those who were able to understand the risk - benefit profile of the assessments and provided written informed consent were included in the study. This study was carried out in accordance with the ethical standards of the responsible institutional committee and with the Helsinki Declaration.

### 

#### Clinical, laboratory and anthropometric assessments

We collected clinical, and anthropometric data such as age, comorbidities, weight, height, body mass index (BMI), fasting glucose, and blood pressure. A single examiner performed the anthropometric measurements, in a standardized way (average of two or more measurements). Weight (kg) was acquired using a precision balance and height was measured using a wall - mounted stadiometer. BMI was calculated as the ratio between weight (kg) and height squared (m^2^), and defined the following reference values: normal (18.5 – 24.9 kg / m^2^), overweight (25.0 – 29.9 kg / m^2^), and obesity (30 kg / m^2^ or higher).

#### LUTS assessment

LUTS were assessed using the International Prostate Symptom Score (IPSS) (10). LUTS severity was classified as follows: mild (IPSS ≤ 7 points), moderate (IPSS ≥ 8 and ≤ 19 points), and severe (IPSS ≥ 20 points). The OAB - V8 questionnaire (Overactive Bladder - Validated 8 - question Screener) was also used to estimate the prevalence of overactive bladder symptoms, which were defined by a score ≥ 8 points (11).

#### Ultrasound assessment

Ultrasound examination of the lower urinary tract was performed with the device Siemens Sonoline G50® (Siemens AG, Munich, Germany). Total thickness of the bladder wall was measured by a mean of two sagittal measurements of the anterior bladder wall, with 250 mL of bladder filling (12, 13). Bladder wall thickness (BWT) was defined by the distance between the mucosa and the adventitia, both with hyperechogenic characteristics (14). Parameters such as prostate gland volume and intravesical prostatic protrusion (IPP) were also evaluated, according to the technique described by Yuen et al. (15). All measurements were performed transabdominally, by a single trained researcher using a high frequency transducer (7.5 MHz). All ultrasound assessments were performed in the urodynamics unit, which allowed measurements with standardized bladder filling (250 mL).

#### Urodynamic assessment

Urodynamic studies were performed 2 to 3 weeks before open prostatectomy, using the Laborie Dorado KT® device (Laborie Medical, Ontario, Canada). All assessments were performed by a single trained researcher, in compliance with the International Continence Society (ICS) Good Urodynamic Practices (16). Post void residual (PVR) was defined as the volume of urine inside the bladder at the end of micturition (17). In our study, PVR was measured by catheterization after free uroflowmetry (before starting the filling cystometry). Increased PVR was arbitrarily defined as a volume ≥ 50 mL (18).

Bladder outlet obstruction (BOO) was defined by the formula: detrusor pressure at maximum flow - (2x maximum flow). A value greater than 40 was regarded as BOO, less than 20 as no obstruction, and between 20 and 40 as undetermined (19). The Schaefer's nomogram was used to assess BOO severity (19). Severe BOO was defined as zone V or VI on the nomogram. Detrusor underactivity was defined by the bladder contractility index (BCI), calculated by the formula: detrusor pressure at maximum flow + (5 x maximum flow). Values under 100 were regarded as detrusor underactivity (19).

#### Bladder biopsies

A full - thickness fragment of the bladder wall measuring 1.0 cm^2^ was obtained from the anterior bladder wall during prostatectomy for determination of OS markers, including catalase, SOD and MDA. In order to aim at the detrusor muscle, each fragment had the mucosa and the perivesical fat removed, and then was frozen in liquid nitrogen at – 70^o^C in order to preserve the material for later analysis.

#### Oxidative Stress Analysis

Prior to performing OS analyzes, the bladder fragments were manually homogenized with 1.15% KPi buffer (pH = 7.4) containing protease inhibitors, at a ratio of 5 mL buffer (1.15% KCl) for each gram of tissue, and then the total protein concentrations of the bladder tissue were measured by the Bradford method (20) in a spectrophotometer at 535 nm.

Thiobarbituric acid reactive substances test was performed by spectrophotometry at 535 nm to assess the concentration of MDA, which is a biomarker of peroxidative damage to lipids (21). Analysis of the activity of antioxidant enzyme SOD was performed by the pyrogallol autoxidation method (22), using spectrophotometry at 420 nm. Determination of catalase activity, which is an antioxidant enzyme, was carried out by the rate of hydrogen peroxide (H_2_O_2_) decomposition (spectrophotometry at 240 nm) (23).

#### Definition of increased oxidative stress

Increased OS was defined by either of the following criteria: increased concentration of MDA and / or decreased concentration of antioxidant enzymes (SOD and / or catalase).

### Statistical analysis

Data were expressed as mean ± standard deviation. Results were compared using the Student t test, and controlled for the use of alpha - blockers. Nominal variables were analyzed using the Fisher exact test. Bonferroni adjustment has been used for multiple testing correction. For specific parameters, such as prostate volume and BWT, distinct quartiles were taken to compare OS levels and define cutoffs. Statistical analyses were performed using SPSS® version 22.0 for Windows (SPSS Inc., Chicago, IL, USA) and an alpha error inferior to 5% (p < 0.05) was considered statistically significant.

## RESULTS

Thirty - eight consecutive patients were included. Mean age was 66.36 ± 6.44 years. Mean body mass index (BMI) was 26.36 ± 2.98 kg / m^2^. The most common comorbidities were systemic arterial hypertension (50%) and diabetes mellitus type 2 (DM2) (29%). Regarding LUTS severity, 14 patients (36.8%) presented mild symptoms, 18 (47.3%) moderate and 6 (15.7%) severe symptoms. Sixteen patients were taking alpha - blockers regularly in the last 6 months before surgery. Prevalence of overactive bladder symptoms (OAB - V8 score ≥ 8 points) was 36.8% (n = 14). Baseline characteristics are described in Table-1.

**Table 1:** Baseline characteristics of men aged ≥ 50 years with LUTS and undergoing open prostate surgery*.

		Mean ± SD
**Clinical parameters**		
	Age (years)	66.36 ± 6.44
	Weight (kilograms)	77.71 ± 8.07
	BMI (kg/m^2^)	26.36 ± 2.98
	IPSS score	11.05 ± 8.72
	OAB-V8 score	7.69 ± 8.44
**Ultrasound parameters**		
	Prostate volume	77.7 ± 20.63
	IPP¥	1.54 ± 0.64
	BWT	3.99 ± 1.39
**Urodynamic parameters**		
	First desire (mL)	195.62 ± 78.57
	Maximum cistometric capacity	360.11 ± 82.05
	(mL)	
	Compliance (mL/cmH_2_O)	79.70 ± 171.72
	BOOI	63.59 ± 31.76
	BCI	108.64 ± 27.33

* n = 38,

¥ n = 17

**BMI =** Body mass index; **IPSS =** International Prostate Symptom Score; **OAB-V8 =** Overactive Bladder-Validated 8-question Screener Questionnaire; **IPP =** Intravesical prostate protrusion; **BWT =** Bladder wall thickness; **BOOI =** Bladder outlet obstruction index; **BCI =** Bladder contractility index

Mean preoperative PSA was 8.17 ± 3.55 vs. 4.48 ± 3.14 ng / mL in patients undergoing open radical prostatectomy (n = 34) and open retropubic prostatectomy (n = 4), respectively (p = 0.16). There were no statistically significant differences in baseline characteristics between the two groups (p > 0.05). All 4 patients who underwent open simple suprapubic prostatectomy had pathological examination confirming BPH. All patients who underwent open radical prostatectomy had low - grade (Gleason 3 + 3 or 3 + 4) organ - confined prostate cancer and concomitant BPH in the pathological examination.

Mean prostate volume estimated by transabdominal ultrasound was 77.7 ± 20.63 cm^3^. Mean bladder wall thickness (BWT) was 3.99 ± 1.39 mm. Seventeen patients (44.7%) presented with intravesical protrusion of the median prostatic lobe, with a mean of 1.54 ± 0.64 cm.

In regards to urodynamics, 13 patients (34.2%) had increased bladder sensation and 7 patients (18.4%) presented reduced bladder sensation. Twelve patients (31.6%) had reduced bladder compliance and 7 patients (18.4%) had reduced cystometric capacity. Detrusor overactivity was demonstrated in 11 patients (28.9%) and urgency urinary incontinence in 5 (13.2%). In the flow - pressure study, reduced peak urinary flow (< 15 mL / sec) was observed in 35 patients (92.1%). Increased post - void residual urine (> 50 mL) was observed in 22 patients (57.9%). BOO was observed in 29 patients (76.3%). Detrusor underactivity was present in 17 patients (44.7%).

### 

#### Primary endpoint

**Figure 1:**
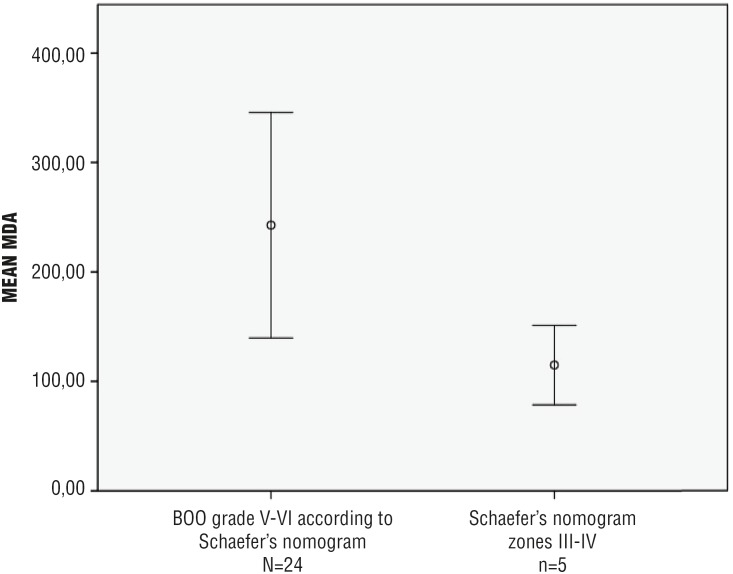
Severity of bladder outlet obstruction according to the Schaefer's nomogram (Zones V-VI versus III-IV) and MDA concentration in the bladder wall of men aged ≥ 50 years, presenting with LUTS and undergoing open prostatectomy (n = 29)*. **MDA =** malondialdehyde; **LUTS =** lower urinary tract symptoms * p = 0.022

Severe BOO (zones V and VI on Schaefer's nomogram) was associated with increased MDA concentration in the bladder wall (242.74 ± 220.20 vs. 114.90 ± 54.08 pmoL / mg; p = 0.022) (Figure-1).

#### Exploratory endpoints

Concentration of OS markers (MDA, SOD, catalase) in the bladder wall according to preoperative parameters (clinical, ultrasound and urodynamic findings) are listed in Table-2.

**Table 2:** Clinical, ultrasound and urodynamic parameters versus oxidative stress markers (MDA, SOD, catalase) in the bladder wall of men aged ≥ 50 years with LUTS and undergoing open prostate surgery*.

Preoperative parameters		Oxidative stress markers (Mean ± SD; p value¥)		
		MDA concentration (pro-oxidant) pmoL/mg	SOD activity (antioxidant enzyme) Usod / mg	Catalase activity (antioxidant enzyme) pmoL/mg
**Clinical**				
**Obesity (BMI** ≥ **30kg/m^2^)**				
	Yes (n = 4)	216.41 ± 199.62	0.4 ± 0.19	170.88 ± 27.46
	No (n = 34)	120.97 ± 101.70	0.76 ± 0.59	317.11 ± 268.98
		p = 0.38	p = 0.05	p = 0.01
**LUTS severity**				
	IPSS≥20 points (n=6)	290.93 ± 237.87	0.69 ± 0.56	181.81 ± 48.59
	IPSS<8 points (n=14)	111.93 ± 82.37	0.71 ± 0.95	327.06 ± 215.17
		p = 0.031	p = 0.48	p = 0.68
**Overactive bladder symptoms**				
	OAB-V8 ≥ 8 points (n = 14)	158.16 ± 133.98	0.62 ± 0.39	314.37 ± 354.72
	OAB-V8 < 8 points (n = 24)	217.88 ± 211.43	0.74 ± 0.63	281.83 ± 138.66
		p = 0.33	p = 0.46	p = 0.74
**Ultrasound**				
**Bladder wall Thickness**				
	≥ 3 mm (n = 30)	342.03 ± 317.03	0.59 ± 0.35	262.15 ± 143.57
	< 3 mm (n = 8)	157.97 ± 107.47	0.69 ± 057	283.47 ± 125.18
		p = 0.015	p = 0.53	p = 0.7
**Prostate volume**				
	≥ 80 cm^3^ (n = 13)	236.43 ± 217.75	0.54 ± 0,31	259.69 ± 102.19
	< 80 cm^3^ (n = 25)	130.33 ± 66.44	0.75 ± 0.61	269.70 ± 157.18
		p = 0.048	p = 0.18	p = 0.81
**IPP**				
	Yes (n = 17)	248.74	0.51 ± 0.29	261.38 ± 125.29
	No (n = 21)	136.37	0.81 ± 0.65	270.27 ± 152.60
		p = 0.064	p = 0.095	p = 0.84
**Urodynamics**				
**Reduced bladder sensation**				
	Yes (n = 7)	202.90 ± 192.37	0.7 ± 0.4	205.66 ± 60.21
	No (n = 31)	155.06 ± 106.91	0.67 ± 0.56	280.30 ± 148.67
		p = 0.44	p = 0.85	p = 0.045
**Urgency** §				
	Yes (n = 13)	250.35 ± 245.70	0.62 ± 0.44	245.68 ± 130.89
	No (n = 25)	166.14 ± 133.19	0.76 ± 0.68	304.03 ± 150.45
		p = 0.28	p = 0.51	p = 0.25
**Detrusor overactivity**				
	Yes (n = 11)	198.57 ± 206.02	0.65 ± 0.43	262.84 ± 125.15
	No (n = 27)	194.73 ± 175.64	0.73 ± 0.74	267.60 ± 146.64
		p = 0.96	p = 0.73	p = 0.92
**Severity of bladder outlet obstruction**				
**according to the Schäfer nomogram** ¶				
	Zones V and VI (n = 24)	242.74 ± 220.20	0.53 ± 0.29	227.75 ± 65.1
	Zones III-IV (n = 5)	114.90 ± 54.08	0.78 ± 0.64	288.89 ± 159.75
		p = 0.022	p = 0.12	p = 0.13
**Detrusor underactivity**				
	Yes (BCI < 100) (n = 17)	222.90 ± 241.21	0.55 ± 0.36	237.39 ± 120.64
	No (BCI ≥ 100) (n = 21)	171.83 ± 107.60	0.77 ± 0.63	290.66 ± 151.38
		p = 0.44	p = 0.19	p = 0.24
**Post-void residual urine**				
	≥ 50 mL (n = 22)	270 ± 197.06	0.48 ± 0.29	212.71 ± 99.32
	< 50 mL (n = 16)	143.96 ± 154.91 p = 0.045	0.97 ± 0.67 p = 0.005	344.61 ± 154.01 p = 0.003

**MDA =** malondialdehyde; **SOD =** superoxide dismutase; **LUTS =** lower urinary tract symptoms; **SD =** standard deviation; **BMI =** body mass index; **IPSS =** International Prostate Symptom Score; **OAB-V8 =** Overactive Bladder-Validated 8-question Screener Questionnaire; **IPP =** intravesical protrusion of the median prostatic lobe; **BCI =** bladder contractility index;

* **n** = 38;

¥ = T test;

§ “urgency” was defined as “the complaint of a sudden compelling desire to pass urine which is difficult to defer” (18);

¶ primary endpoint

Obesity was associated with reduced activity of the antioxidant enzyme catalase (170.88 ± 27.46 vs. 317.11 ± 268.98 pmoL / mg in obese and in non - obese patients, respectively; p = 0.01) and with reduced activity of the antioxidant enzyme SOD (0.4 ± 0.19 vs. 0.76 ± 0.59 Usod / mg in obese and in non - obese patients, respectively; p = 0.05).

Patients with severe LUTS (IPSS score ≥ 20 points) had higher MDA concentration in the bladder wall when compared to the patients with mild LUTS (IPSS < 8 points): 290.93 ± 237.87 vs. 111.93 ± 82.37 pmoL / mg, respectively (p = 0.031). Patients with moderate LUTS (IPSS ≥ 8 and < 20 points) had a mean of MDA concentration of 144.85 ± 112.88 pmoL / mg (intermediate values in - between mild and severe LUTS outcomes, but not statistically significant in the multivariate analysis). Likewise, the diagnosis of OAB (OAB - V8 score ≥ 8 points) had no association with increased OS in the bladder wall (p > 0.05).

MDA concentration was higher in patients with BWT ≥ 3 mm compared to those with BWT < 3 mm (342.03 ± 317.03 vs. 157.97 ± 107.47 pmoL / mg, respectively; p = 0.015). Increased prostate volume (PV) was associated with higher concentrations of MDA: 236.43 ± 217.75 vs. 130.33 ± 66.44 pmoL / mg in patients with PV ≥ 80 cm3 and PV < 80cm3, respectively (p = 0.048). However, IPP was not associated with increased OS in the bladder wall (p > 0.05).

Regarding urodynamic parameters, it has been shown that patients with reduced bladder sensation had a statistically significant reduction of the activity of the catalase enzyme in the bladder wall when compared to those patients with normal bladder sensation (205.66 ± 60.21 vs. 280.30 ± 148.67 pmoL / mg, respectively; p = 0.045). On the other hand, parameters such as urinary urgency, detrusor overactivity, reduced bladder compliance or cystometric capacity, and weak stream were not associated with increased OS in the bladder wall (p > 0.05).

Increased MDA concentration and reduced activity of antioxidant enzymes (both catalase and SOD) were observed in the bladder wall of patients with post - void residual urine (PVR) ≥ 50 mL:

MDA: 270 ± 197.06 vs. 143.96 ± 154.91 pmoL / mg; p = 0.045 (Figure-2a),

**Figure 2a:**
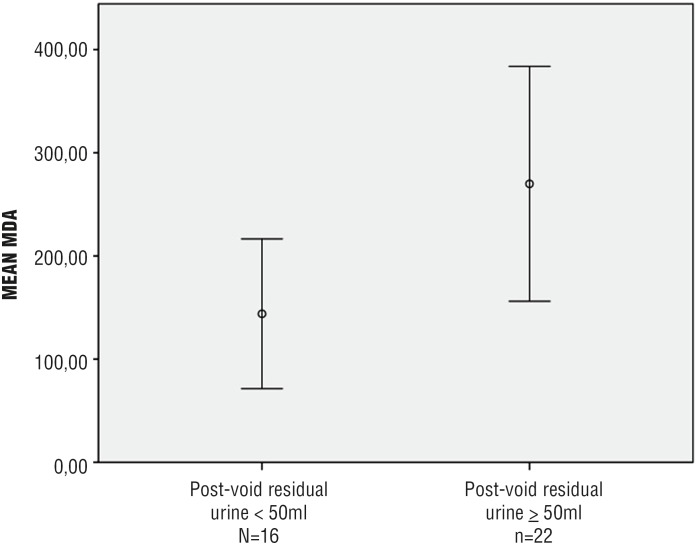
Increased MDA concentration in the bladder wall of men aged ≥ 50 years, undergoing open prostatectomy and presenting with LUTS and PVR ≥ 50 mL (n = 38)* **MDA =** malondialdehyde; **LUTS =** lower urinary tract symptoms; **PVR =** post-void residual urine * p = 0.045

SOD: 0.48 ± 0.29 vs. 0.97 ± 0.67 Usod / mg; p = 0.005 (Figure-2b),

**Figure 2b:**
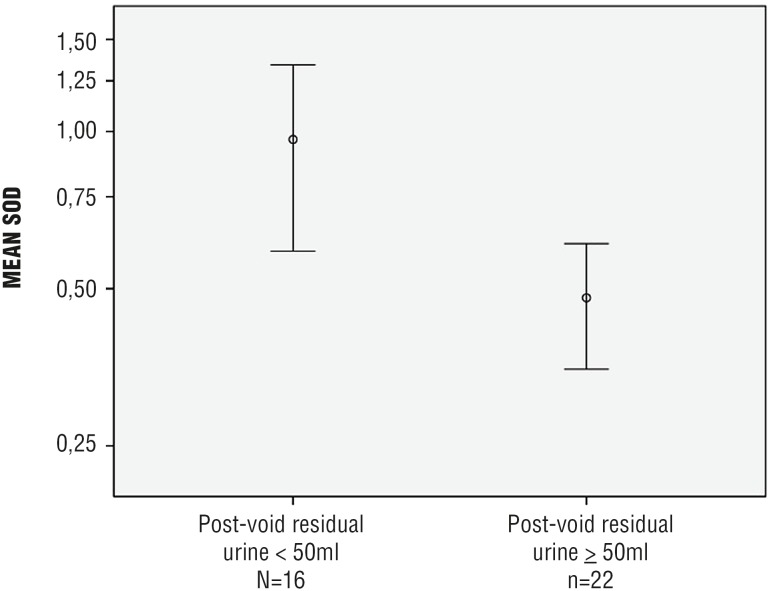
Decreased activity of the antioxidant enzyme SOD in the bladder wall of men aged ≥ 50 years, undergoing open prostatectomy and presenting with LUTS and PVR ≥ 50 mL (n = 38)* **SOD =** superoxide dismutase; **LUTS =** lower urinary tract symptoms; **PVR =** post-void residual urine * p = 0.005

Catalase: 212.71 ± 99.32 vs. 344.61 ± 154.01 pmoL / mg; p = 0.003 (Figure-2c).

**Figure 2c:**
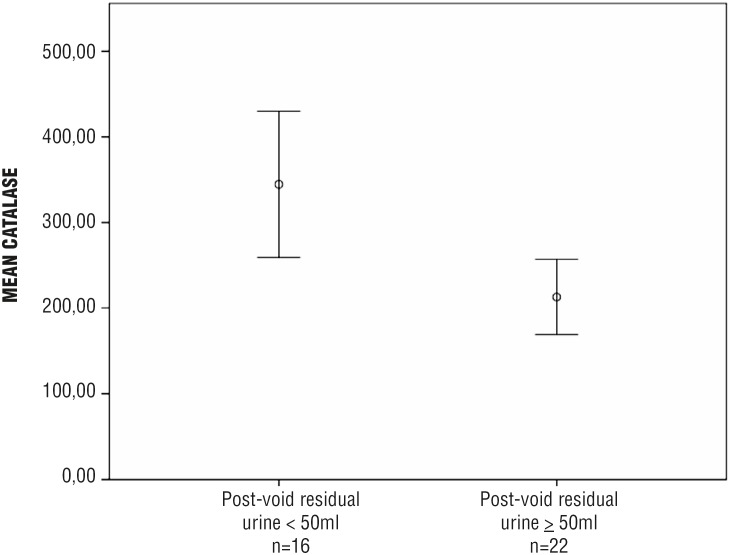
Decreased activity of the antioxidant enzyme catalase in the bladder wall of men aged ≥ 50 years, undergoing open prostatectomy and presenting with LUTS and PVR ≥ 50 mL (n = 38)* **LUTS =** lower urinary tract symptoms; **PVR =** post-void residual urine * p = 0.003

## DISCUSSION

To our knowledge, this is the first study investigating OS markers (MDA, SOD and catalase) in the detrusor muscle of humans undergoing open prostate surgery. Clinical factors (LUTS severity and obesity, ultrasound findings (bladder wall thickness ≥ 3 mm, prostate volume ≥ 80 cm3), and urodynamic parameters (BOO severity, post - void residual urine ≥ 50 mL) were associated with either increased MDA concentration or reduced activity of antioxidant enzymes (SOD / catalase) in the bladder wall. Identification of such factors may have clinical relevance, as evidence from animal models suggested a relationship between increased OS and bladder dysfunction (24-26).

Generation of reactive oxygen species (ROS) and ischemia - reperfusion injury have been proposed as the primary etiological factors in obstruction - induced bladder dysfunction (9). At a molecular level, reactive oxygen species exhibit signaling and cell - function - modifying roles (27). OS occurs when the net flux of reactive oxygen species (ROS) and reactive nitrogen species (RNS) production exceeds the capacity of the cell to detoxify these potentially injurious oxidants. Functional in vitro studies showed that elevated ROS levels impair bladder contractile responses (28, 29).

Several biomarkers have been used in experimental models to assess both urinary and plasma OS, including 8 - hydroxy – 2’ - deoxyguanosine (8 - OHdG), MDA, total anti - oxidant capacity (TAC) and glutathione (GSH) (30). SOD and catalase assays have also been carried out on animal tissues to study the effects of partial BOO on the cell's anti - oxidant defense mechanisms (25). In our pilot study, OS biomarkers included MDA, catalase and SOD. Experimental studies showed that both the SOD and catalase activities are calcium - sensitive and changes in their activity would be expected to occur during ischemia, which can result in decreased antioxidant capacity of the bladder smooth muscle and mucosa (31). Hence, there sensitivity and specificity issues related to distinct OS biomarkers and caution should be exercised in interpreting the results of such studies.

There is growing interest on the association between LUTS and systemic conditions such as obesity, diabetes mellitus and metabolic syndrome in men (32, 33). Dibello et al. (33) compared the prevalence of metabolic syndrome in men aged ≥ 50 years with and without a diagnosis of BPH from a large database (UK Clinical Practice Research Datalink - CPRD). Among men with BPH, 26.5% (n = 85.103) had diagnosis of metabolic syndrome, compared with 20.9% without BPH (control group, n = 85.103) (absolute difference of 5.6%, p < 0.001). In our pilot study, data on height and weight of the patients were recorded and allowed the calculation of BMI. Patients with BMI ≥ 30 (n = 4) presented with higher levels of both antioxidant enzymes catalase and SOD in the bladder wall in comparison with those with BMI < 30 (n = 34).

Non - invasive assessment of PVR is usually recommended as a first - line diagnostic tool in men with LUTS (34). Despite the lack of a consensual cutoff, it is known that increased PVR may represent a risk factor for acute urinary retention and / or bladder dysfunction among these patients (35). According to Crawford et al., who studied a total of 3.047 men with BPH over 4.5 years, a baseline PVR of 39 mL or greater was an independent predictor of BPH clinical progression in patients not receiving active treatment for LUTS (36). Our study showed that a PVR ≥ 50 mL was associated with increased OS in the bladder wall (higher MDA concentration; reduced activity of both catalase and SOD).

Although not recommended in the routine assessment of LUTS in men without neurological diseases, urodynamics may be important for selected patients in order to define the pattern of voiding dysfunction (34). Incomplete bladder emptying may be caused by detrusor underactivity (DU), bladder outlet obstruction (BOO), or dysfunctional voiding (17, 37). Previous studies showed DU in up to 40% of men aged > 65 years and even up to 48% of men aged ≥ 70 years (38, 39). Despite being a prevalent condition in the elderly population, the origin of DU in men without neurological diseases remains controversial (40). BOO and increased OS have been related to a higher risk of detrusor underactivity in animal models (25, 41, 42). Callaghan et al. demonstrated that partial BOO has significant effects on the activity of both SOD and catalase in the bladder, with variations that are dependent on the severity and duration of the obstruction (25). Our study demonstrated that patients with severe LUTS (IPSS ≥ 20 points; n = 6) and severe BOO (grade V - VI in Schaefer's nomogram; n = 24) had increased MDA concentration in the bladder wall in comparison with patients with mild LUTS (IPSS < 8 points; n = 14) and BOO grade III - IV (n = 5), respectively. These findings may be clinically relevant, as bladder dysfunction has been implicated as a risk factor for persistent LUTS after prostate surgery (2).

Other non - invasive tests, such as ultrasound measurement of IPP, detrusor wall thickness (DWT) or bladder wall thickness (BWT) have been advocated to predict the chances of BOO in the male population (12, 37). Increased DWT is observed in adult men with non - neurogenic LUTS and BOO (12). A prospective study demonstrated that DWT ≥ 2 mm in bladders filled ≥ 250 mL was a predictor of BOO (43). Additionally, Güzel et al. have studied the utility of BWT in men with HPB / LUTS and demonstrated that this parameter is an easy, quick, and repeatable test to predict BOO severity (44). Most probably, due to the high proportion of patients with BOO in our study, we could not demonstrate an association between BWT and obstruction. On the other hand, BWT ≥ 3 mm was associated with increased OS in the bladder wall (p = 0.015).

The main limitation of our pilot study is the small number of patients included. Since the introduction of 5 - alpha - reductase inhibitors in the clinical practice, the number of patients who require open prostate surgery for BPH has dropped significantly (45). On the other hand, BPH and prostate cancer (PCa) are often coexisting in older men (46). Bocking et al. (47) demonstrated that latent prostate cancer would be present in more than 50% of men over the age of 80. In our study, most of the patients underwent open radical prostatectomy (n = 34); notwithstanding, all of them had been followed up at the urology outpatient clinic of the same institution due to BPH symptoms prior to the diagnosis of prostate cancer. In addition, all patients who underwent open radical prostatectomy had low - grade (Gleason 3 + 3 or 3 + 4) organ - confined prostate cancer and concomitant BPH in the pathological examination. Another limitation of our study was the lack of OS analysis in the urothelial layer of the bladder samples. Evidence from animal models suggests that the urothelium is also sensitive to oxidative stress generated by partial obstruction (48). However, as per protocol, our study was aimed at assessing the oxidative stress in the smooth muscle layer (detrusor). In addition, despite the use of previously validated assessment procedures, there is no perfect method for assessing tissue OS. To counteract oxidative and nitrosative stress, human cells employ complex defense mechanisms (27). The interpretation of all OS studies should be done in the light of such imperfection, since there are distinct cellular enzymatic and non - enzymatic antioxidative pathways. At last, the invasiveness of bladder biopsies may also be seen as a limitation. Future research should focus on non - invasive techniques and biomarkers to assess the oxidative stress in the human bladder.

Despite inherent limitations, our pilot study has several strengths. Firstly, our restricted inclusion criteria aimed at patients with higher risk of BOO (76.3% of our study population). Secondly, the technique for retrieving bladder biopsies in our protocol did not require electrocautery and favored the OS analysis. Thirdly, ultrasound and urodynamic assessments were standardized and performed by a single trained researcher, who was blind to the OS analysis and results.

## CONCLUSIONS

This pilot study revealed increased MDA concentration in the bladder wall of men with severe LUTS (IPSS score ≥ 20 points), severe BOO (Schaefer's nomogram grade V - VI), BWT ≥ 3 mm and PV ≥ 80 cm3. Reduced activity of the antioxidant enzymes (catalase and / or SOD) was demonstrated in patients with BMI ≥ 30 and in those with reduced bladder sensation on filling cystometry. Increased PVR (≥ 50 mL) was associated with both increased MDA concentration and reduced activity of antioxidant enzymes (catalase and SOD) in the bladder wall.

Novel diagnostic methods targeting oxidative and / or inflammatory pathways may be a reasonable strategy for a more comprehensive evaluation of patients presenting with severe LUTS and BOO. Further studies are still needed to assess the role of non - invasive biomarkers of OS in predicting bladder dysfunction in men with LUTS.
